# Poster Session II - A242 COMORBIDITY BURDEN AND MORTALITY IN PEOPLE WITH INFLAMMATORY BOWEL DISEASE

**DOI:** 10.1093/jcag/gwaf042.242

**Published:** 2026-02-13

**Authors:** S Okabayashi, S Coward, J W Windsor, L Hracs, J Gorospe, G G Kaplan

**Affiliations:** University of Calgary, Calgary, AB, Canada; University of Calgary, Calgary, AB, Canada; University of Calgary, Calgary, AB, Canada; University of Calgary, Calgary, AB, Canada; University of Calgary, Calgary, AB, Canada; University of Calgary, Calgary, AB, Canada

## Abstract

**Background:**

As inflammatory bowel disease (IBD) undergoes an epidemiologic shift toward an aging population, the prevalence of comorbidities and their impact on mortality among people with IBD remain poorly understood.

**Aims:**

To evaluate changes in comorbidity patterns and their association with mortality over two decades using a population-based IBD cohort.

**Methods:**

A retrospective cohort study was conducted using population-based health administrative data from Alberta (2002–2021); including 42,903 prevalent and 21,587 incident cases. Fifteen chronic conditions, including cancers, ischemic heart disease, chronic heart failure, hypertension, diabetes, asthma, chronic pulmonary disease, osteoarthritis, rheumatoid arthritis, osteoporosis, mood and anxiety disorders, dementia, Parkinson’s disease, stroke and transient ischemic attack, and chronic kidney disease were identified with validated algorithms (listed in figure 1) and mortality was identified using Vital Statistics data. Mortality was age and sex standardized to the 2011 Canadian population. Rates (per 1000 person-years) were calculated using the IBD population as denominators. Standardized mortality ratios (SMR) were calculated through comparison of IBD and Canadian population mortality rates. Average annual percent change (AAPC), with 95% confidence interval (CI), was calculated using log-linear regression. Rate ratios (RR) comparing mortality between comorbidity groups (0, 1, and ≥2 comorbidities) were calculated using Poisson regression. Age- and sex- adjusted hazard ratios (aHR), with 95%CI, of the five-year mortality of incident cases comparing 0, 1, and ≥2 comorbidities was calculated using Cox proportional hazard models.

**Results:**

Comorbidities among people with IBD increased from 44.8% in 2002 to 86.0% in 2021, with mental health disorders, cancer, osteoarthritis, and hypertension being the most common. Mortality in IBD did not significantly change overtime (AAPC −0.20; 95%CI: −1.16, 0.77), and the SMR was 1.20 (95%CI: 1.15, 1.25). Mortality rates (per 1000 person-years) increased with the increase in number of comorbidities (no comorbidities: 0.47; 1 comorbidity: 1.74; ≥2 comorbidities: 18.4). For those with 1 or ≥ 2 comorbidities, compared to those with no comorbidity, had a significantly higher rate of mortality with an RR of 4.00 (95%CI: 2.70, 5.94), and 82.4 (95%CI: 58.9, 115.3), respectively. In incident cases, having an increased number of comorbidities at diagnosis was significantly associated with 5-year mortality (1 comorbidity vs none: aHR 2.59 [95% CI: 1.96, 3.42]; ≥2 comorbidities vs none: aHR 8.10 [95% CI: 6.26, 10.5]).

**Conclusions:**

Multimorbidity has become increasingly prevalent among people with IBD and represents a major determinant of mortality, highlighting the importance of comprehensive care strategies to improve prognosis in IBD.

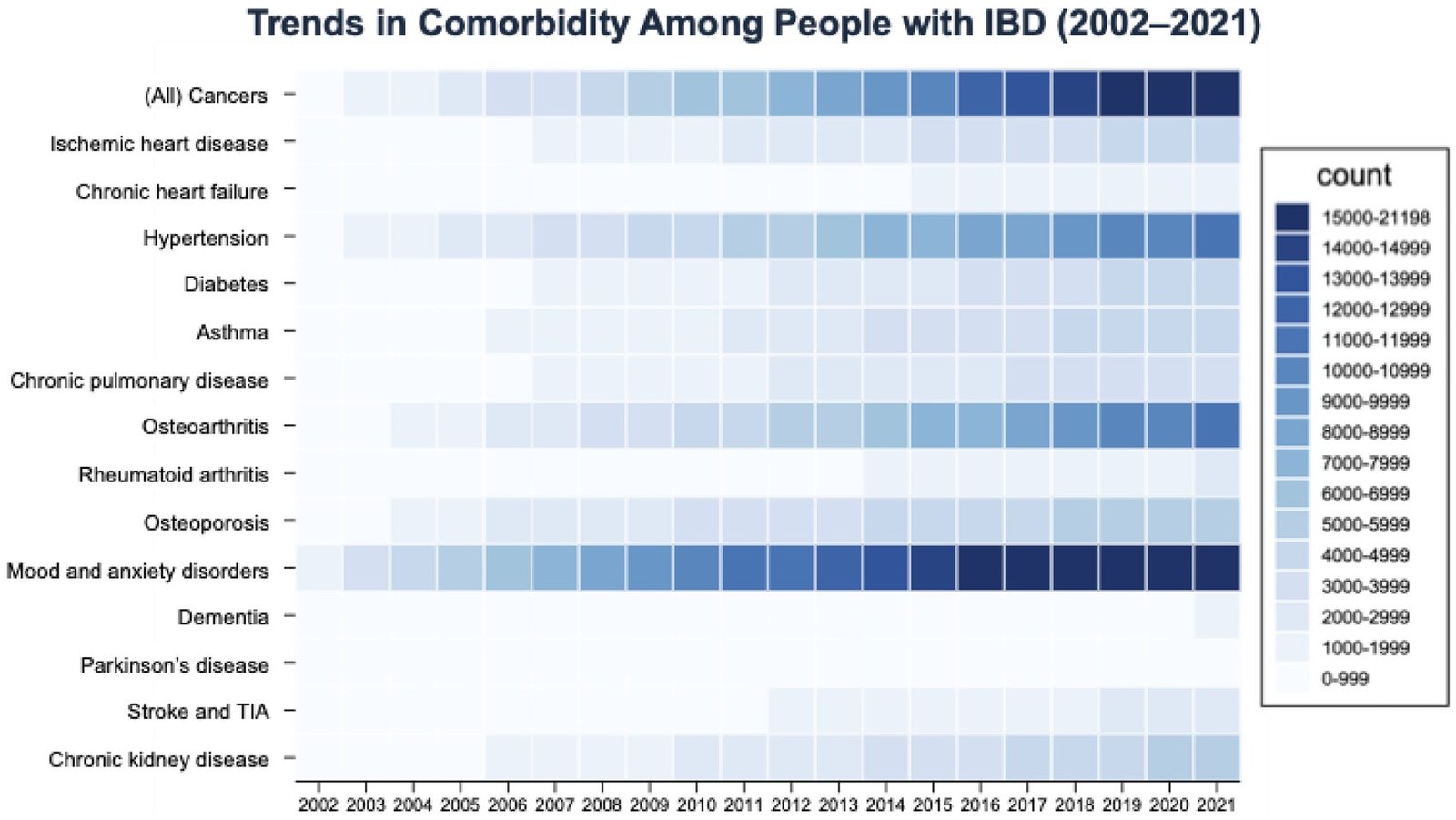

**Funding Agencies:**

The Alberta Innovates Postdoctoral Fellowships Program (AI PDF)

